# Prevalence, aetiology and host and management factors associated with bovine mastitis in dairy cows in Zoba Anseba, Eritrea: a cross-sectional study

**DOI:** 10.1186/s12917-025-04868-9

**Published:** 2025-07-02

**Authors:** Redie Kidane Ghebrehawariat, Zerai Woldehiwet, Tzeggai Tesfai, Bereket Habte Imam, Yirgalem Mehari Kibrom, Abraham Debru Habte, Betiel Habte Hadgu, Filmon Berhane Kahsay, Rim Berhane Fisehaye, Samuel Haile Kahsay, Saron Yemane Yosief

**Affiliations:** 1 School of Veterinary Medicine, Hamelmalo Agricultural College, Hamelmalo, Eritrea; 2 Livestock Division, National Agricultural Research Institute, Halhale, Eritrea; 3 National Animal & Plant Health Laboratory, Asmara, Eritrea; 4 Institute of Infection, Veterinary and Ecological Sciences, University of Liverpool, Liverpool, UK

**Keywords:** Prevalence, Risk factors, Bovine mastitis, Cross-sectional study, Eritrea, California mastitis test (CMT), Antimicrobial resistance, *Staphylococcus aureus*, *Streptococcus agalactiae*, *Enterococcus faecium*

## Abstract

From 47 dairy farms located in the Anseba Region of Eritrea, 222 cows were randomly selected for a questionnaire survey, examined for signs of clinical mastitis and milk samples tested for subclinical mastitis using the California mastitis test (CMT). Twenty cows (9%) were deemed to have clinical mastitis (CM), because they had visible clinical signs or visible changes in milk samples. Of the 202 cows tested by the CMT, 156 (77.23%) had a score of 1 or above at least in one quarter and were deemed to have subclinical mastitis (SCM).

SCM was significantly more frequent in exotic breeds than in cross or indigenous breeds (*p* < 0.001) and cows with poor body condition compared to cows with good body condition (*p* = 0.025). CM was significantly more frequent in cows with a history of clinical mastitis (*p* = 0.004). SCM was significantly more frequent in farms that keep dung inside the compound (*p* = 0.004), do not milk cows with mastitis last (*p* < 0.01), use earthen floor (*p* < 0.01), do not routinely use mastitis test (*p* < 0.001), have intensive system of production and in farms with frequent tick infestation of the udder (*p* < 0.001).

From 20 CM-positive and 153-SCM-positive composite milk samples tested, 29 isolates of *Staphylococcus aureus*, 23 *Enterococcus faecium* and 20 *Streptococcus* species, including 8 *Streptococcus agalactiae* were isolated. Of the 13 isolates of *S. aureus* tested, all were resistant to tetracycline, 11 to sulfonamides and 10 to penicillin. Two *Streptococcus agalactiae* isolates tested were resistant to tetracycline and penicillin.

**Supplementary Information:**

The online version contains supplementary material available at 10.1186/s12917-025-04868-9.

## Introduction

Bovine mastitis is an inflammation of the mammary glands, primarily caused by invading pathogens [1], but extrinsic and intrinsic factors can also have an influence on the disease [2]. Due to the high costs of treatment and control, reduction in the yield and quality of milk and early culling of cows, bovine mastitis accounts for a significant portion of the economic loss encountered by the dairy industry [3]. Various host risk factors such as breed, age, lactation stage, history of clinical mastitis, management factors and methods of milking have been associated with the occurrence of bovine mastitis [4].

This disease is of particular concern in countries such as Eritrea, where there are limited resources for animal health and poor management and milking practices. We are not aware of any published information about the prevalence of mastitis in dairy cows or other ruminants in Eritrea but several reports from regions of Sudan and Ethiopia, which have similar climates and systems of dairy management indicate high prevalence of clinical and subclinical mastitis [5, 6]. For example, a study on 400 lactating cows in smallholder dairy farms in Northern State of Sudan, found a prevalence of 10.5% for clinical mastitis (CM) and a prevalence of 72% for subclinical mastitis (SCM), at cow level [5]. Several studies conducted in small-scale and large-scale dairy farms of Ethiopia reported prevalence of CM ranging between 3 and 21 2.7–21.0% and prevalences of 33.3 to 68.1% for SCM [6–9]. The study carried out in Sudan found an association between SCM and barn size, use of and frequency of removing bedding, washing hands before milking, source of water, frequency of dung removal and milking cows with mastitis last [5]. Several studies in Ethiopia identified host factors such as breed, age, parity and stage of lactation, and management factors including herd size, type of floor, washing hands before milking each cow to be potential risk factors for CM or SCM [6–9].

The main objective of the present study was to estimate the prevalence of CM and SCM and to identify possible host and management risk factors in 47 dairy herds in Zoba Anseba, Eritrea.

## Materials and methods

### Description of study area

**Fig. 1:**
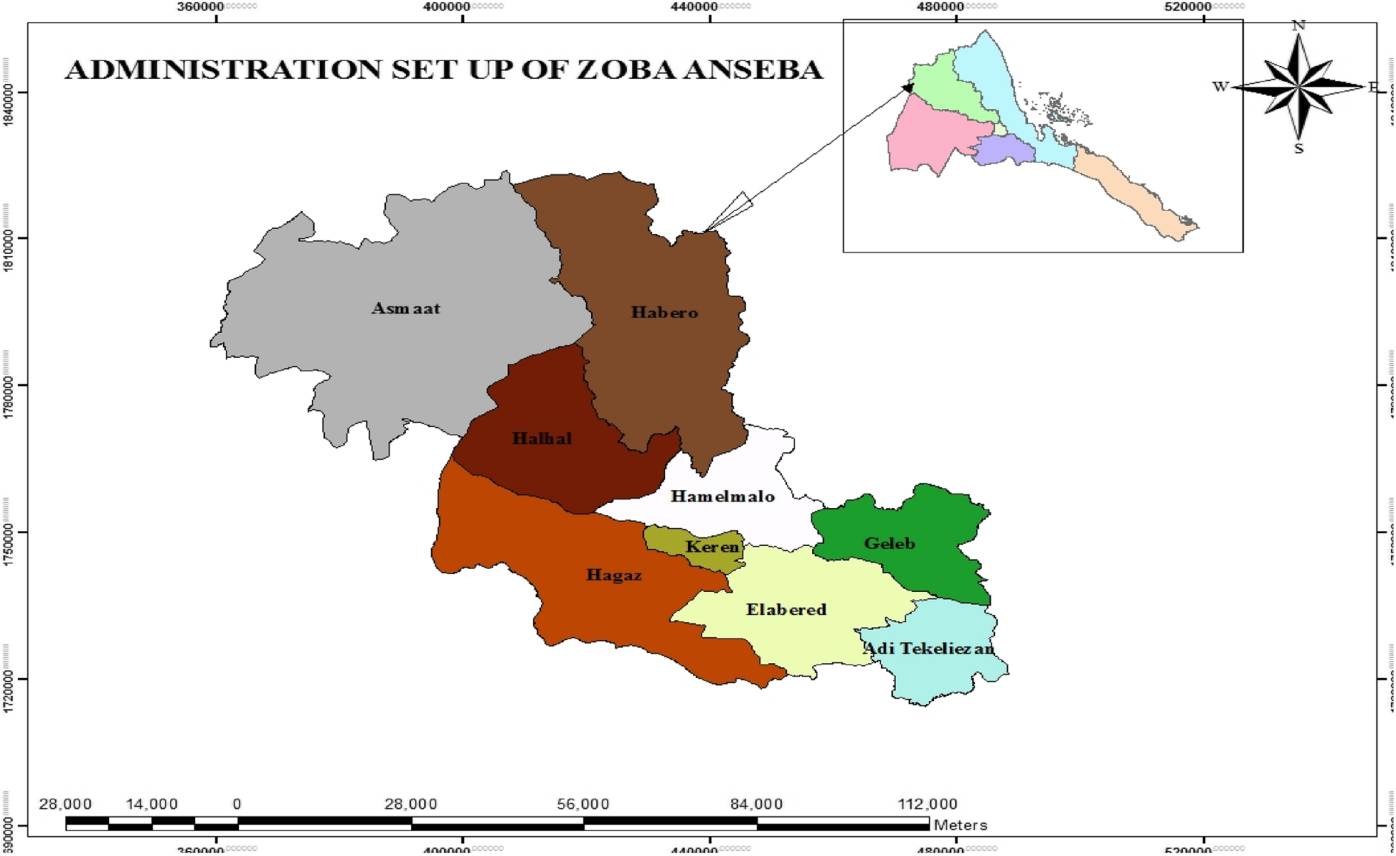
Map of Eritrea showing the study areas in Zoba Anseba

The cross-sectional study was conducted in Zoba Anseba, subzones Hamelmalo, Keren, Hagaz, Elabered and Aditekelezan (Fig. 1). Zoba Anseba was selected as a good area to undertake such a study because it is one of the main regions of dairy production, with many dairy herds located near Hamelmalo Agricultural College (HAC), an institution with expertise and adequate facilities for such a study.

### Study design and sample size

All techniques involving animals and their care adhered to the ethical guidelines of international standards and was approved by the Ethical Review Committee of HAC and the National Agricultural Research Institute (NARI) and the Ministry of Agriculture (MOA). The sample size was determined according to the formula given by Thrusfield [10] as follows.

$$\mathrm n\;=\;\frac{1.96^2\;{\mathrm P}_{\exp}\left(1-{\mathrm P}_{\exp}\right)}{\mathrm d^2}$$

Where n = required sample size, P_exp_ = Expected prevalence, d = desired absolute precision.

Hence, the sample size was calculated at 95% statistical confidence level, 5% precision, and with an expected prevalence of 85% from a previous study in Ethiopia [11], thus yielding a sample size of 195 lactating cows.

### Questionnaire survey

A semi-structured questionnaire on potential host and management factors for bovine mastitis was conducted by face-to-face interviews of dairy farm owners or managers. Host factors included breed, body condition score, age, parity, lactation period, presence of udder or teat injury, previous history of mastitis and presence of milk leaking from one or more teats. Cows with good body condition were those with good fat covers. Those with medium body condition were those with less fat cover and with visible skeletal structures and cows that were emaciated and with visible skeletal structures were described as having poor body condition.

The management factors in the questionnaire included herd size, type of flooring (concrete or earthen), disposal of dung, presence of roofing, use of bedding, methods of milking (squeezing or stripping), washing hands before milking each cow, washing udder before milking, use of separate clean towel after washing, dipping teats after milking, regular testing of cows for mastitis, milking cows with mastitis last, use of dry-cow therapy, culling cows with recurrent mastitis and presence of ticks on the udder. One person, owner or manager, from each farm responded to the questionnaire. The questionnaire data is presented as Supplementary Material 1.

### Selection of study animals

The cross-sectional study was carried out in 47 dairy farms randomly selected from 63 farms located in Zoba Anseba Eritrea. Any dairy farm in the region with at least one lactating cow had an equal chance of being included in the study. Of the 47 herds randomly selected from the 63 herds in the study area, only 4 herds had more than 17 lactating cows, one with 93 lactating cows, one with 29 lactating cows and 2 herds with 19 lactating cows each. Milk samples were collected from half of the lactating cows randomly selected from each herd, unless the cow was at early stage of parturition or at late stage of pregnancy (Supplementary Material 1). The 47 herds were managed under semi-intensive or intensive systems. On intensive farms, cattle were kept indoors all the time and given roughage and concentrates. The semi-intensive farms were characterized by outdoor grazing during the day. The cows used in the study included 186/Holstein, 29 cross-bred and 7 indigenous breeds.

### Collecting milk samples

All milk samples were collected between 27 July and 4 August 2022.

Before collecting milk samples for CMT and for bacterial isolation, each cow was physically examined for the presence of classical clinical signs of mastitis, swelling, heat and redness [12]. In addition, tick infestation, atrophy or blockage of teats were recorded. After cleaning and drying the udder and disinfecting the teats, the first three streams of milk were discarded and abnormalities in milk including the presence of flakes, blood, clots, and watery secretion recorded. Milk samples were then aseptically collected from each quarter into a sterile 5 mL tube.

### California mastitis test

Milk samples from each quarter were tested for mastitis using CMT according to the supplier instructions. Briefly, after putting 2 mL of milk from each teat into a separate paddle, an equal amount of CMT reagent was added. After gentle rotation of the mixture in a horizontal direction, changes in the milk’s fluidity and increased viscosity related to high somatic cell count (SCC) and mastitis were recorded and graded according to Quinn et al. [13]. Cows with at least one quarter having a CMT score of 1 and above were judged to have SCM. Composite milk samples from cows with CM and cows judged to have SCM were placed in a cold chain and sent to the National Animal and Plant Health Laboratory (NAPHL) for bacteriological examination.

### Determination of clinical and subclinical mastitis

Cows with CM were those with classical clinical signs of mastitis and the secretion of abnormal milk such as the presence of blood, clots, or other changes in milk samples. Because of the serious constitutional changes in the milk, CMT score results of cows with CM were not used for further analysis. Cows without clinical signs but with a CMT score of 1, 2 or 3 in at least one quarter were deemed to have SCM.

### Isolation and identification of pathogenic bacteria

Composite milk samples from 20 cows with clinical mastitis and 153 cows with at least one quarter having a CMT score of 1 or above were sent to the NAPHL for bacterial isolation and identification according to the method of Cowan and Steel [14]. Gram-positive, catalase-positive cocci from beta haemolytic colonies were characterized as *Staphylococcus aureus* based on mannitol fermentation on mannitol salt agar and the production of coagulase [15]. Briefly, isolates suspected as *S aureus,* based on colony characteristics, Gram stain, and catalase reaction, were inoculated onto selective mannitol salt and incubated at 37 °C for 24 h. Isolates that produced colonies surrounded by yellow zones in this medium and a positive coagulase reaction were considered as *S. aureus*. All catalase-negative cocci were classified as *Streptococcus* species or *Enterococcus faecium* based on haemolytic patterns of their colonies on blood agar and using a commercial streptococcal grouping kit (Oxoid, UK).

### Susceptibility to antibiotics

The disk diffusion assay was used to test selected isolates of *Staphylococcus aureus*, *Enterococcus faecium, Streptococcus agalactiae, S. dysgalactiae* and *S. canis* for their susceptibility to sulfonamide, tetracycline, penicillin, chloramphenicol, streptomycin, oxytetracycline, neomycin, and gentamicin. They are the most widely used antibiotics in the country. For this purpose, a portion of a single colony was selected from the overnight bacterial culture using sterile swab and diluted in normal saline to give a turbidity of 0.5 McFarland Standard (equivalent to 1–5 × 10^6^ CFU ml − 1). Muller Hinton agar plates were swabbed with bacterial culture to cover the entire plate before adding 4 antibiotic discs. After 24 h incubation at a temperature of 37 °C during 24 h the diameter of growth inhibition zone was measured by caliper in millimeters and compared with the standard guidelines to categorize the isolate as susceptible, moderately susceptible, or resistant.

#### Analysis of data

Questionnaire data and CMT results were recorded and coded using Microsoft Excel. Cows with clinical signs of mastitis or changes to milk were considered to have CM and cows with at least one quarter recording a CMT score of 1 or more were deemed to have SCM. Herd and cow level prevalence of SCM and CM and quarter level prevalence of CMT scores were calculated by dividing the number of positive by the total number of herds, cows, or quarters. Host and management risk factors associated with SCM and CM were analyzed by SPSS 23.0 (SPSS Inc, Chicago, Illinois, USA). The risk factors were tested for association with SCM and CM using the Pearson chi-square test and by calculating the odds ratio (OR). The *p*-value of < 0.05 was considered to be statistically significant. The logistic regression model of multivariate analysis was used to investigate possible association between management risk factors and the probabilities of the occurrence of SCM, using management risk factors, which had high OR scores and found to be significantly associated with SCM, based on the Chi-square test, as independent variables.

## Results

### Prevalence of clinical and subclinical mastitis

Based on clinical signs and changes in the character of the milk in one or more quarters, 20 cows were deemed to have CM. Because of the serious constitutional changes in the milk, CMT score results of cows with CM were not used for further analysis.

Of the 202 cows tested by CMT, 156 (77.23%) had a score of 1 or above at least in one quarter and were deemed to have SCM.

### Host risk factors and their association with bovine mastitis

Pearson’s chi-square test and cross-tabulation analysis was used to establish the association of host and management risk factors for subclinical and clinical mastitis. At cow level, breed was found to be significantly associated with SCM, with significantly more cases of SCM in Holsteins than in other breeds (*p* < 0.001, Table 1). However, of the 222 cows used in the study 186 were Holstein, 29 were cross-bred and only 7 were indigenous. SCM was also significantly more frequent in cows with poor body condition compared to those with good body condition (p < 0.025). History of mastitis was significantly associated with CM (*p* = 0.004, odds ratio (OR) = 3.99) but not with SCM (Table 1). There was no statistically significant association of age, parity, and lactation period with SCM or CM (Tables 1 and 2).

**Table 1:** Association of host factors with SCM

Variable	Category	Negative	Positive	Pearsons 2-sided		Odds ratio	CI at 95%	
				X^2^	p		Lower	Upper
Breed								
	Holstein	46	140	21.934	< 0.001	NA		
	Cross	13	16					
	Indigenous	7	0					
Body condition								
	Good	21	63	7.397	0.025	NA		
	Medium	31	80					
	Poor	14	13					
Udder Injury								
	Yes	58	146	2.53	0.154	2.01	0.76	1.28
	No	8	10					
History of mastitis								
	Yes	58	133	2.05	0.606	0.56	0.24	1.28
	No	8	23					
Milk Leakage								
	Yes	2	1	1.988	0.159	0.21	0.02	2.32
	No	64	155					
Parity								
	5 to 9	18	36	0.444	0.505	0.80	0.41	1.54
	1 to 4	48	120					
Lactation month								
	1 to 3	21	44	1.245	0.505	NA		
	4 to 6	24	50					
	7 plus	21	62					
Age of cow								
	3 to 6	38	92	0.037	0.847	1/06	0.59	1.89
	7 to 12	28	64					

*NA* not applicable

**Table 2:** Association of host factors with clinical mastitis

Variable	Category	Negative	Positive	Pearsons		Odds ratio	CI at 95%	
				X^2^	*P value*		Lower	Upper
Breed								
	Holstein	169	17	4.00	0.819	NA		
	Cross	27	2					
	Indigenous	6	1					
Body condition								
	Good	79	5	2.031	0.362	NA		
	Medium	98	13					
	Poor	25	2					
Udder Injury								
	Yes	187	17	1.401	0.237	0.15	0.03	0.69
	No	15	3					
History of mastitis								
	Yes	24	7	8.096	0.004	3.99	1.45	10.99
	No	178	13					
Milk Leakage								
	Yes	3	0	0.301	0.583	NA		
	No	199	20					
Parity								
	5 to 9	47	7	1.361	0.243	1.78	0.67	4.72
	1 to 4	155	13					
Lactation month								
	1 to 3	60	5	0.196	0.907	NA		
	4 to 6	67	7					
	7 plus	75	8					
Age of cow								
	3 to 6	122	8	3.120	0.077	0.31	0.12	0.79
	7 to 12	80	12					

*NA* not applicable

### Management factors and their association with bovine mastitis

All but 3 of the owners or managers were males, 21 had no formal education, 6 had elementary education, 16 had secondary education and 5 had completed university education (Supplementary Material 1). The number of cows included from each farm varied from 1 to 41, depending on the size of the farm and the number of lactating cows available at the time of sampling (Supplementary Material 1). Except for one large herd, which had 93 lactating cows, the number of lactating cows was very small. At the time of sampling, 35 herds had not more than 10 lactating cows, with 20 herds having less than 5 lactating cows each. Ten herds had 11 to 19 lactating cows each and one herd had 29 lactating cows. The mean number of cows selected from the 46 small herds was 4.7 ± 5.9 per farm (Supplementary Material 1).

**Fig. 2:**
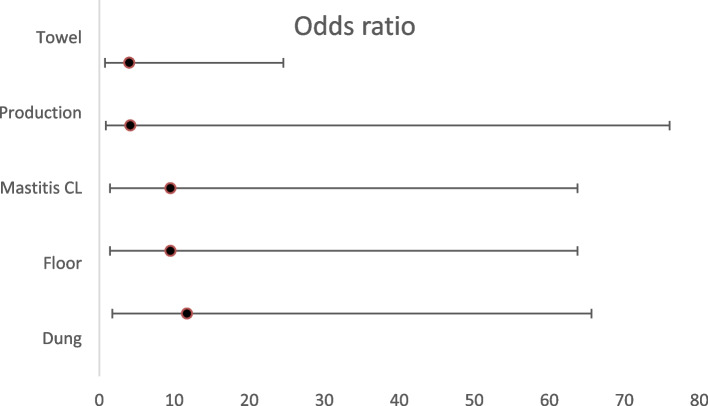
Forest plot of odds ratio of 5 management risk factors associated with SCM at farm level. Use of Towel: No or Yes. Production: intensive or semi-intensive. Mastitis CL: Milking cows with mastitis with other cows or at the end. Floor: earthen or concrete. Dung: kept inside or outside compound

SCM was detected in one or more cows randomly selected from 42 herds, but CM was detected only in 14 herds, (Tables 3 and 4), with two herds each having 4 cases of CM. The manager’s gender, age, or level of formal education had no significant association with SCM or CM on the cows they manage. Management factors, such as dipping after milking, use of roofing and bedding were not analyzed because they were used only by very few farms. However, several management factors were found to have statistically significant association with SCM at herd level (Table 3; Fig. 2) and at cow level (Tables 5 and 6). At herd level, the management factors which were significantly associated with SCM were keeping dung inside the compound (*p* = 0.004, OR = 11.67), not milking cows with mastitis last (*p* = 0.001, OR = 9.25), not using mastitis test routinely (*p* = 0.001, OR = 40), use of earthen floor compared to concrete floor (*p* = 0.01, OR = 9.25) and frequent or occasional presence of ticks on the udder and teats of cows (*p* < 0.01). There was a statistically significant association between the production system and SCM (*p* = 0.04, OR = 7.81), with cows under intensive production systems being more likely to have SCM.

**Table 3:** Association of management factors with SCM at farm level

Variable	Category	Negative	Positive	Pearsons		Odds ratio	CI at 95%	
				X^2^	*P* value		Lower	Upper
Dung disposal								
	In	2	35	8.46	0.004	11.67	1.74	78.44
	Out	4	6					
Floor								
	Earthen	3	37	6.69	0.01	9.25	1.39	62.09
	Concrete	3	4					
Mastitis cow last								
	No	3	37	6.69	0.01	9.25	1.39	62.09
	Yes	3	4					
Mastitis test								
	No	3	40	15.206	< 0.001	40.00	3.13	511.90
	Yes	3	1					
Ticks on udder								
	Frequently	0	13	18.385	< 0.001	NA		
	Rarely	2	26					
	Never	4	2					
Production								
	Intensive	1	25	4.157	0.04	7.81	0.83	73.13
	Semi-intensive	5	16					
Dry-cow therapy								
	No	5	37	0.263	0.608	1.85	0.17	20.24
	Yes	1	4					
Towel used								
	No	2	28	2.771		4.31	0.70	26.61
	Yes	4	13	0.09				
One towel for 1 cow								
	No	4	17	1.345	0.246	0.35	0.06	2.13
	Yes	2	24					
Wash udder								
	No	0	3	0.469	0.493	NA		
	Yes	6	38					
Clean floor								
	Weekly	0	4	0.64	0.424	NA		
	Daily	6	37					
Culling								
	No	6	38	0.457	0.499	NA		
	Yes	0	3					
Herd Size								
	< 10	4	15	1.967	0.161	0.29	0.05	1.70
	> 10	2	26					
Roof								
	No	5	1	1.133	0.287	0.31	0.03	2,90
	Yes	25	16					

*NA* not applicable

**Table 4:** Association of management factors with CM at farm level

Variable	Category	Negative	Positive	Pearsons 2-sided		Odds ratio	CI at 95%	
				X^2^	*P* value		Lower	Upper
Dung disposal								
	In	25	12	0582	0.466	1.92	0.35	4.22
	Out	8	2					
Floor								
	Earthen	29	11	0.672	0.402	1.98	0.38	10.29
	Concrete	4	3					
Milk mastitis cow last								
	No	29	12	0.06	0.939	0.97	0.16	5.69
	Yes	5	2					
Mastitis test								
	No	30	14	1.797	0.180	NA		
	Yes	4	0					
Ticks on udder								
	Frequently	11	2	6.122	0.047	NA		
	Rarely	16	12					
	Never	6	0					
Production								
	Intensive	18	8	0.027	0.870	1.11	0.31	1.17
	Semi-intensive	15	6					
Towel used								
	No	17	13	7.277	0.007	12.24	1.43	104.96
	Yes	16	1					
One towel for 1 cow								
	No	17	5	0.649	0.421	1.91	0.53	6.94
	Yes	16	9					
Udder washed								
	No	2	1	0.029	0.89	1.19	0.99	14.30
	Yes	31	13					
Floor cleaned								
	Weekly	2	2	0.917	0.338	2.67	0.22	32.06
	Daily	32	12					
Culling								
	No	31	13	0. 019	0.890	0.81	0.05	9.73
	Yes	2	1					
Herd size								
	< 10	13	6	0.049	0.825	1.15	0.32	4.10
	> 10	20	8					
Dry cow therapy								
	No	29	13	0.256	0.613	1.79	0.18	17.65
	Yes	4	1					
Roof								
	No	22	8	0.386	0.534	1.5	0.42	5.41
	Yes	11	6					

*NA* not applicable

**Table 5:** Association of management factors with SCM at cow level

Variable	Category	Negative	Positive	Pearsons		Odds ratio	CI at 95%	
				X^2^	*P* value		Lower	Upper
Dung disposal								
	In	31	108	9.818	0.002	2.54	1.49	3.43
	Out	35	48					
Floor								
	Earthen	29	74	0.228	0.633	1.15	0.65	2.05
	Concrete	37	82					
Mastitis cow last								
	No	48	118	0.209	0.648	1.16	0.61	2.24
	Yes	18	38					
Mastitis test								
	No	51	130	1.131	0.287	1.48	−0.28	0.88
	Yes	15	26					
Ticks on udder								
	Frequently	10	16	1.449	0.485	NA		
	Rarely	35	94					
	Never	21	46					
Production								
	Intensive	15	70	9.625	0.002	2.77	1.44	5.34
	Semi-intensive	51	86					
Dry-cow therapy								
	No	41	112	2.026	0.155	1.55	0.85	2.85
	Yes	25	44					
Towel used								
	No	21	35	2.1644	0.098	0.62	0.33	1.18
	Yes	45	121					
One towel for 1 cow								
	No	43	122	4.144	0.042	1.92	1.02	3.61
	Yes	23	34					
Wash udder								
	No	3	2	2.244	0.134	0.27	0.04	1.67
	Yes	63	154					
Clean floor:								
	Weekly	1	8	1.556	0.212	3.51	0.43	28.67
	Daily	65	148					
Culling								
	No	63	141	1.600	0.206	0.45	0.13	1.60
	Yes	3	15					
Herd Size								
	= < 10	46	122	1.924	0.177	1.56	0.82	4.82
	> 10	20	34					
Roof								
	No	29	74	0.228	0.633	0.87	0.44	0.72
	Yes	37	82					

*NA* not applicable

**Table 6:** Summary output of logistic regression with disposal of dung, production system and use of towel to clean udder as independent variables and SCM as the dependent variable

**Variables in the Equation**									
Step 0	Variables	Score	df	Sig					
	Disposal of dung	9.818	1	0.002					
	Production system	9. 625	1	0.037					
	Use of towel	2.164	1	0.141					
	Overall statistics	18.708	3	< 0.001					
Step 1	Variables						95% CI for Exp{B)		
		B	SE	Wald	df	Sig	Exp{B)	Lower	Upper
	Dung disposal	.966	.326	8.753	1	.003	2.627	1.385	4.981
	Production type	.789	.349	5.108	1	.002	2.201	1.110	4.362
	Use of towel	-.606	.357	2.875	1	.090	.546	.271	1.099
	Constant	.207	.248	.692	1	.405	1.230		

The management factors with statistically significant association with CM, at herd level, were not using a towel to dry the udder (*p* = 0.007, OR = 12.24) and presence of ticks on the udder (*p* = 0.05, Table 4).

At cow level, a significant association was observed between SCM and keeping dung inside the compound (*p* = 0.002, OR = 2.54), intensive system of production (*p* = 0.002, OR = 2.77), and not using a separate towel to dry each udder (*p* = 0.042, OR = 1.92, Table 5). Logistic regression was carried out using 3 management factors which were shown to have significant effect on SCM at cow level as independent variables and SCM as the dependent variable. The model showed significant improvement in goodness of fit compared to the null model (*p* < 0.001). The Hosmer and Lemeshow test confirmed that the model fits the data well, with no or very little difference between the observed and expected. According to this model, keeping dung inside the compound and intensive production system were the most important management risk factors with *p* = 0.003 and *p* = 0.002 respectively (Table 6). The model correctly predicted 70.3% of cases, with 98.7% sensitivity of predicting SCM-positive cases.

### Bacteriological examination

Nine of the 20 composite milk samples obtained from cows with CM and 71 of the 153 composite milk samples obtained from cows with SCM yielded pure growth of bacteria or fungi after 24 h incubation at 37 °C. The predominant isolates were staphylococci, streptococci, and enterococci (Table 7). The most dominant pathogen was *Staphylococcus aureus,* with 4 isolates from cows with CM and 25 cows with SCM*. Enterococcus faecium* (Lancefield Group D) was detected in 3 cows with CM and 17 cows with SCM. *Streptococcus agalactiae* (Lancefield Group B) was isolated from a cow with CM and 7 cows with SCM. *Streptococcus canis* (Lancefield Group G) was isolated from 4 cows with SCM. *Streptococcus pyogenes* (Lancefield Group A) and *Streptococcus faecalis* (Group D) were each isolated from 3 composite milk samples obtained from cows with SCM. *Streptococcus dysgalactiae* (Lancefield Group C) and *Streptococcus porcinus* (Lancefield Group E) were each detected in 2 cows with SCM and one cow with SCM yielded growth of *S. qui* (Lancefield Group C). There was a statistically significant association between the isolation of a pathogen from a composite milk sample and the number of CMT-positive quarters (*p* < 0.003, Table 8).

**Table 7:** Staphylococci and streptococci isolated from composite milk samples obtained from cows with CM with SCM

Pathogen	CM	SCM	Haemolysis	Catalase	Coagulase	Ferment mannitol	Lancefield group
*S. aureus*	4	25	Beta	+ ve	+ ve	+ ve	NA
CNS	1	1	-ve	+ ve	-ve	-ve	NA
*S. agalactiae*	1	7	Beta	-ve	NA	NA	B
*S. canis*	0	4	Beta	-ve	NA	NA	G
*S. pyogenes*	0	3	Beta	-ve	NA	NA	A
*S. faecalis*	0	3	Gamma	-ve	NA	NA	D
*S. dysgalactiae*	0	2	Alpha	-ve	NA	NA	C
*S. porcinus*	0	2	Beta	-ve	NA	NA	E
*S. equi*	0	1	Beta	-ve	NA	NA	C
*Enterococcus faecium*	3	17	Alpha	-ve	NA	NA	D

*CNS* coagulase negative staphylococci, *NA* not applicable, + *ve* positive, *-ve* negative

**Table 8:** Association of number of CMT-positive quarters and bacterial isolation in composite milk sample

CMT-positive quarters	IMI- positive	IMI-negative	Pearsons X2	*P* value
1	6	19	14.138	0.003
2	12	19		
3	14	22		
4	39	22		

*IMI* intramammary infection

### Resistance to antibiotics

All of the 13 isolates of *S. aureus* tested were resistant to tetracycline, 11 were resistant to sulfonamides and 10 to penicillin. The number of *Streptococcus agalactiae* isolates tested was small (only 2) but both were resistant to sulfonamide, tetracycline, and penicillin (Table 9). Four of the 6 isolates of *Enterococcus faecium* tested were resistant to tetracycline and sulfonamides and 2 were resistant to penicillin (Table 9).

**Table 9:** Resistance of *Staphylococcus aureus*, *Enterococcus faecium* and *Streptococcus* species isolated from cows with CM or SCM against commonly used antibiotics

Antimicrobial	*S. aureus*	*E. faecium*	*S. agalactiae*	*S. dysgalactiae*	*S. canis*	*S. pyogenes*	*S. porcinus*
Sulfonamide	11	4	2	1	1	1	0
Tetracycline	13	4	2	1	2	1	1
Penicillin	10	2	2	1	1	0	1
Streptomycin	5	1	0	1	1	0	0
Chloramphenicol	4	0	0	0	0	0	0
Oxytetracycline	1	1	1	0	0	0	0
Neomycin	0	0	0	0	0	0	0
Gentamycin	0	0	0	0	0	0	0
Total tested	13	6	2	2	3	1	1

## Dsiscussion

Mastitis is widely recognized as one of the most economically important diseases hampering the dairy industry. In most developed economies, as clinical mastitis caused by contagious pathogens became under control, subclinical mastitis caused by environmental pathogens became the main problem [16]. Well-managed dairy farms also have the resources and obligations to have their milk samples routinely tested for mastitis and SCC. The CMT is now widely used as a standard test for SCM [17]. In countries with poor animal health provisions and poor milking practices, both CM and SCM are known to be major obstacles to dairy milk production [6]. The control of bovine mastitis in dairy farms depends mainly on good milking and other management practices, including treatment of cows with CM, good drying-off practices to reduce incidences of SCM and reducing host and management risk factors associated with mastitis [18].

The prevalence and the pathogenic agents associated with bovine mastitis in Eritrea are not known and there is no published data on the subject. In the present study, the prevalence of SCM was estimated to be 70.27%, with cases of CM based on clinical signs were 9%. Similar to the prevalence levels were previously described in Sudan [5], Ethiopia [6, 19] and in Pakistan [20], countries with similar socioeconomic conditions.

Two of the host factors that were found to be associated with SCM in the present study were breed and body condition. Previous studies in Ethiopia have shown that poor body condition in dairy cows is strongly linked to a higher prevalence of mastitis [21, 22]. Cows with lower body condition scores are more susceptible to mastitis because poor nutrition can weaken their immune response to infectious agents [21].

There were significantly more cases of SCM in Holstein breeds compared to indigenous breeds or mixed breeds. However, because the number of cross and indigenous breeds used in the present study was small, further studies with more herds with indigenous and cross = breeds are necessary to establish the effects of breed with CM or SCM. The present study also indicated that SCM was significantly associated with poor body condition. Studies in Ethiopia and Kenya have reported similar findings [6, 23, 24]. This is also in agreement with other reports indicating that poor body condition can be a risk factor for bovine mastitis. For example, Locker et al. [25] reported that cows with a low body condition score during lactation were more susceptible to mastitis, and cows with mastitis are likely to have low body condition score.

Several management practices such as milking procedures, housing, bedding, free stall system, frequent use of the CMT, cleaning the calving pen after each calving may have an impact on the prevalence of bovine mastitis [26, 27].

In the present study, keeping cow manure within the compound and housing cows on earthen floors were found to be significantly associated with SCM. Dung or cow manure and muddy earth are known to be the main sources of the most common environmental pathogens of bovine mastitis. To reduce the load of these pathogens in the cows’ environment, it is important to reduce organic contamination by frequent manure removal, by avoiding overstocking and providing clean, dry bedding [28]. Several studies in Ethiopia have found significant association between bovine mastitis and host factors such as breed, lactation stage, history of mastitis, floor type, and teat injury and management factors such as size of herd, milking cows with mastitis last and type of bedding used [6, 21, 23].

In the present study, the frequent presence of ticks on the udder was found to be significantly associated with CM at the herd level. Similar observations were also reported in studies carried out in Ethiopia [29] and in Zimbabwe [30]. Of the 48 farms used in the present study, only 6 confirmed that they never noticed ticks on the udder of their cows. The damage caused by tick infestation can significantly influence the development of CM by allowing bacterial invasion on the teats damaged by tick bite [31]. The association of mastitis with ticks may also be related to an immunosuppression caused by tick-borne pathogens. For example, it has been known for a long time that cows and small ruminants infected by the tick-borne pathogen *Anaplasma phagocytophilum* suffer from reduced milk yield and mastitis and are prone to other secondary bacterial and viral infections as the pathogen infects and reduces the phagocytic ability of neutrophils [32, 33].

The other management factors with statistically significant association with CM were not using a towel to dry the udder and the presence of ticks on the udder.

The present study found that all farmers used hand milking and most of them failed to wash their hands and the udder and hand before milking every cow and did not use clean towel to dry the udder. Those management factors might have enhanced the transmission of contagious and environmental pathogens of mastitis. The route of transmission could be between infected and uninfected cows and between infected and uninfected udder quarters during milking.

A combination of regular testing for mastitis and the use of dry cow therapy based on antibiotics or teat-sealants has been shown to reduce SCM during the subsequent lactation [17, 18]. In the present study, only 5 of the 48 farms used dry cow therapy and only 4 farms tested their cows for mastitis.

Most of the pathogenic bacteria isolated from composite milk samples of cows with clinical mastitis and those with CMT scores of 1 or above were staphylococci, streptococci and enterococci. Of the 79 isolates identified as causing intramammary infection, 29 (36.7%) were classified as *Staphylococcus aureus*, 20 (25.3%) as *Streptococcus faecium*, 8 (13.6%) as *Streptococcus agalactiae*, 4 (5.1%) as *Streptococcus canis* and 3 (3.8%) as *Streptococcus pyogenes*. As far as we are aware, this is the first study to document bacterial causes of bovine mastitis in Eritrea. Further studies are currently underway to establish the role of pathogenic bacteria on bovine mastitis in other regions of Eritrea. A recent systemic review of 46 studies in Ethiopia reported that the most common bacterial pathogen isolated from cows with CM or SCM was S. aureus, followed by *S. agalactiae* and *S. dysgalactiae* [34].

Although the number of isolates used was small, the present study revealed that nearly all isolates of *Staphylococcus aureus* from cows with CM or SCM were resistant to tetracycline, sulfonamides, and penicillin. We are not aware of any other study on antimicrobial resistance of *S. aureus* isolated from dairy milk or other sources in Eritrea. However, in other countries, the increasing detection of antimicrobial-resistant strains of *S. aureus* and other bacteria in human, animal, food, and environmental samples has been associated with the extensive use of antimicrobials in livestock in general [35] and the use of antibiotics to treat and prevent mastitis, in particular [36]. It was beyond the scope of the present study to establish the extent of antimicrobial resistance in *S. aureus*, including the possible presence of methicillin-resistant *Staphylococcus aureus* (MRSA) in milk. Therefore, further studies are recommended to establish the extent of antibiotic resistance among strains of *S. aureus* circulating in animal populations in Eritrea in general and in milk in particular. There is no published information about the prevalence of antimicrobial resistance (AMR) in Eritrea. However, a recent systemic review highlighted the alarming increase in the prevalence of AMR among *S. aureus* and other bacterial pathogens in Ethiopia [37]. A study in Khartoum State of Sudan associated the presence of AMR *S. aureus*, *E. coli*, and other bacteria isolated from cows with mastitis with misuse of antibiotics in animal farming, and to be a contributing factor to the emergence and spread of AMR bacteria among the human population [38]. Although originally considered a problem to human health, the emerging crisis of AMR requires a “One Health” approach, encompassing human, animal, and environmental reservoirs [36]. In this regard, the extensive use of antibiotics in the livestock production systems to treat mastitis and other bacterial diseases can lead to the presence of AMR genes in bacteria that contaminate or naturally occur in milk and dairy products, thereby introducing them into the food chain. The preliminary findings of the present study highlight the importance of testing for AMR bacteria in milk and other animal products widely used as human food, as part of a Global Plan on AMR. The latest Joint External Evaluation report on IHR Core Capacities of the State of Eritrea recommended the need to increase surveillance of AMR pathogens in both human and human health sectors [39].

## Conclusion

The results of the present study showed that the prevalence of bovine mastitis in Zoba Anseba, Eritrea, is very high. From the animal risk factors investigated, poor body condition and breed were significantly associated with subclinical mastitis. The association of poor body condition with mastitis highlights the importance of improving the nutrition of lactating animals, not only on improving milk yield but the cow’s well-being too. The tole of breed on mastitis requires further investigation because the number of indigenous breeds used in the present study was very small. The management factors that were significantly associated with SCM and CM observed in the present study were related to poor management and milking practices. To reduce the high incidence of mastitis, relatively simple control measures such as not keeping dung inside, avoiding the use of earthen or muddy flooring, milking cows with mastitis last, and using a clean towel to dry each udder are urgently needed. In addition, the present study showed that SCM was significantly more prevalent in herds with a history of tick infestation of the udder. With only 6 of 47 herds having no history of ticks on the udder of their cows, tick control measures are also urgently required. The bacteriological study carried out in the present investigation showed that most of the bacteria isolated from cows with CM or SCM were those known to be contagious in nature, namely *Staphylococcus aureus*, *Streptococcus agalactiae* and environmental pathogens including *Streptococcus dysgalactiae* and *Enterococcus faecium*. This highlights the importance of good milking and management practices to reduce the spread of infection from one animal to another. The present study also revealed that nearly of the isolates identified as *Staphylococcus aureus* were resistant to tetracycline, penicillin and sulfonamides. This underscores the importance of testing for AMR bacteria in milk and other animal products widely used as human food. It is hoped that the present findings will have a positive influence on local dairy management policies and national mastitis control strategies by encouraging better milking and management practices and regular testing for CM and SCM.

## Supplementary Information

Supplementary Material 1.

## Data Availability

Data that support the findings of the present study are available with the corresponding author upon reasonable request.

## Abbreviations

CFU: Colony-forming unit
CM: Clinical mastitis
CMT: California mastitis test
FAO: Food and Agricultural Organization
HAC: Hamelmalo Agricultural College
IMI: Intramammary infection
MOA: Ministry of Agriculture
MRSA: Methicillin-resistant *Staphylococcus aureus*
NAPHL: National Animal and Plant Health Laboratory
NARI: National Agricultural Research Institute
OR: Odds ratio
SCC: Somatic cell count
SCM: Subclinical mastitis
